# Building cloud computing environments for genome analysis in Japan

**DOI:** 10.1038/s41439-022-00223-8

**Published:** 2022-12-14

**Authors:** Osamu Ogasawara

**Affiliations:** Bioinformation and DDBJ Center, National Institute of Genetics, 1111 Yata, Mishima, Shizuoka 411-8540 Japan

**Keywords:** Genome informatics, Genetic databases

## Abstract

This review article describes the current status of data archiving and computational infrastructure in the field of genomic medicine, focusing primarily on the situation in Japan. I begin by introducing the status of supercomputer operations in Japan, where a high-performance computing infrastructure (HPCI) is operated to meet the diverse computational needs of science in general. Since this HPCI consists of supercomputers of various architectures located across the nation connected via a high-speed network, including supercomputers specialized in genome science, the status of its response to the explosive increase in genomic data, including the International Nucleotide Sequence Database Collaboration (INSDC) data archive, is explored. Separately, since it is clear that the use of commercial cloud computing environments needs to be promoted, both in light of the rapid increase in computing demands and to support international data sharing and international data analysis projects, I explain how the Japanese government has established a series of guidelines for the use of cloud computing based on its cybersecurity strategy and has begun to build a government cloud for government agencies. I will also carefully consider several other issues of user concern. Finally, I will show how Japan’s major cloud computing infrastructure is currently evolving toward a multicloud and hybrid cloud configuration.

## Genomics: Cloud environments for data analysis in Japan

An overview of the computational infrastructure available for genomic research in Japan highlights the importance of establishing hybrid cloud computing platforms. The enormous growth of genomic data necessitates a corresponding increase in computational infrastructure for analyses. A review by Osamu Ogasawara of Japan’s National Institute of Genetics, Shizuoka, Japan, highlights the strengths and challenges in the country’s computational infrastructure. Several powerful supercomputers are linked to universities by a high-speed network for transferring large data sets. Commercial cloud services also provide on-demand availability, easy international collaboration, and reproducible workflows. However, the cloud market is dominated by three vendors, and there are concerns about privacy and data security. Ogasawara advocates a hybrid cloud approach allowing for on-premise analyses and movement between vendors, and notes that the government is developing a multi-cloud environment for domestic institutes.

## Introduction

Recent advances in measurement technology, including the reduction in cost of sequencing analysis, have made large amounts and varieties of data available to both independent laboratories and specialized research institutions. The successful use of these large amounts of data in research is now seen as crucial to tackling various problems that have been difficult to solve in the past, such as the identification of genes related to hereditary diseases and the early detection and treatment of cancers^1^. However, while the availability of various data has expanded research possibilities, problems related to sharing and analyzing these large-scale data among researchers have emerged as computational infrastructure issues.

For example, when considering only the time required for network communication, it is not realistic to download several hundred terabytes of data to the computers of each laboratory. In addition, each laboratory would face budgetary and security problems related to preparing computer environments capable of storing and analyzing such large amounts of data. Therefore, instead of bringing data to user environments (bringing the data to the people), it is more realistic to bring the user’s analysis environment to the place where the data resides (bringing the people to the data)^1^.

To accomplish this purpose, the use of supercomputers maintained by research institutes or the use of commercial cloud services provides a good starting point. One can also see that the use of whole-genome sequencing (WGS) analysis in research and clinical practice is increasing due to the declining analysis costs of next-generation sequencers. Additionally, even though it was previously believed that the speed of data analysis would be unable to keep up with the increasing speed of sequencers, that problem is being resolved by the spread and advancement of computational infrastructures, such as supercomputers and cloud computing, as well as the development of high-speed software that takes advantage of the special computer hardware.

For example, Clara Parabricks^2^, which is a genome analysis software suite produced by the Nvidia Corporation (NVIDIA), includes a Genome Analysis Toolkit (GATK)^3,4^ with compatible algorithms. When using a calculation node equipped with four NVIDIA V100 general purpose graphic processing units (GPGPUs), the software can analyze 30·WGS data in ~90 min, while eight A100 GPGPUs can process the same data in less than 30 min. This is a substantial reduction from the 30 h of analysis time required when using the official central processing unit (CPU)-only implementation of GATK.

Similarly, the Illumina DRAGEN™ Platform uses highly reconfigurable field-programmable gate array (FPGA) technology, while Sentieon Analysis Pipelines & Tools uses parallel CPU computation to considerably accelerate the processing speed^5,6^. Separately, PEZY Computing has developed a high-speed genome analysis system that runs on PEZY SC2 many-core processors that can complete the alignment process of 30· WGS data within 10 min and the whole process, including a variant call within 15 min^7,8^.

This mini-review describes the current status of data archiving and computational infrastructure that enables “bringing people to the data” style analysis in the field of genomic medicine, focusing primarily on the situation in Japan. I begin with a discussion on the use of supercomputers for genomic medicine in Japan, after which I explore how to use commercial cloud computing services in Japan in light of Japanese laws and guidelines, as well as points to be aware of when using them. Finally, since not all workloads match the “bring people to the data” approach, the status of how to configure hybrid clouds to accommodate a variety of workloads is discussed.

## Use of supercomputers and high-speed networks in genomic medicine research in Japan

### HPCI and the Fugaku supercomputer

A high-performance computing infrastructure (HPCI) was established to provide academics at universities and national institutes with opportunities to perform research using supercomputers^9^. At the center of the HPCI is Fugaku, the world’s highest-performance supercomputer, which was ranked first in computing power in the Top500 supercomputer ranking^10^, the high-performance conjugate gradient (HPCG) benchmark^11^, the Graph500 ranking of supercomputer systems^12–14^, and the high-performance LINPACK—artificial intelligence (HPL-AI) ranking^15^ from June 2020 to November 2021^16^.

**Fig. 1:**
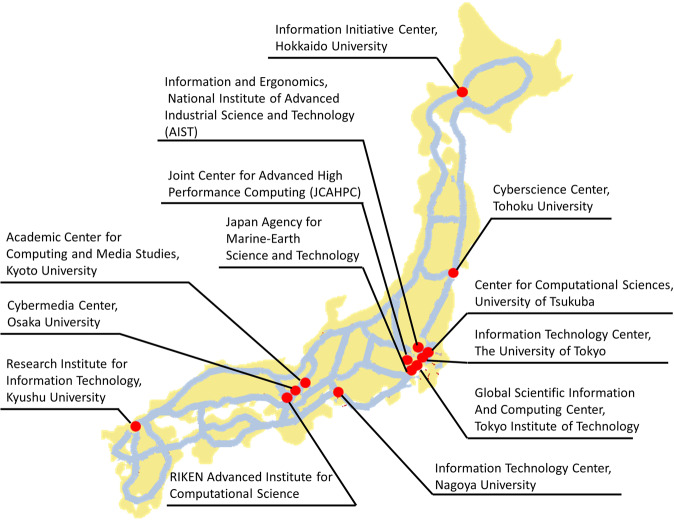
Institutions comprising HPCI and SINET6 backborn network. Blue lines indicate SINET6 400 Gbps backborn network which connects all regions of Japan (excluding Okinawa, which is connected at 100 Gbps). Red dots are the location of HPCI participating institutions (2021). The supercomputer Fugaku, the core of HPCI, is located at RIKEN Advanced Institute for Computational Science.

HPCI resources include a variety of other computers equipped with the same CPU model as Fugaku, as well as computers with Intel and AMD CPUs, computers with calculation accelerators such as GPGPUs and vector processors, and computers with large memory capacities. These supercomputers are all connected to shared storage at high speed (Fig. 1) via the science information network (SINET)^17,18^, and most are available to researchers free of charge^9^, thus making it possible to meet the diverse needs of a wide variety of research fields involving both universities and the private sector.

There is also a project underway aimed at permitting cloud-like use of Fugaku (Fugaku Cloud Platform) to improve its convenience, and empirical studies are being conducted on various usage methods. These include using Fugaku to execute analysis programs serviced by commercial clouds or, conversely, sending computation requests to Fugaku as an extension of researchers’ own computers (on-premise servers, private clouds) in the form of cloud bursts.

### SINET, a high-speed academic information network

Whether downloading large-scale data to one’s own computer or using it on a supercomputer or in a cloud, the availability of a high-speed network is a prerequisite. Hence, funded by the Japanese Ministry of Education, Culture, Sports, Science and Technology (MEXT), the National Institute of Informatics (NII) has developed and now operates SINET, a high-speed academic information network connecting ~1000 universities and private research institutes in Japan. SINET was migrated to a new system (SINET6) in April 2022, which consists of a 400 Gbps backbone network connecting all regions of the country (Fig. 1). The SINET backbone network is also connected to the internet in the US^19^ and GÉANT in Europe^20^ via 100 Gbps lines to facilitate the international distribution of the research information required for advanced research projects. This backbone network is available to all universities free of charge, and prospective institutions can join by subscribing to a trunk line from one of the many data centers positioned at various locations on the network.

Although each participating institution is required to pay a fee for a leased line between itself and the backbone line, the high-speed network with bandwidths available at 10 Gbps base, 100 Gbps, and 400 Gbps levels is available at much lower costs than would be the case if the entire network were built using commercial lines. In addition, by connecting a leased line to a cloud vendor via SINET, a large discount can be obtained on the cloud vendor’s network usage fees. Private companies can also use SINET if they participate in pilot programs or conduct joint research with universities and national research institutes.

This means that Japanese universities and research institutes can enjoy a high-speed network environment that is of a fairly high standard, even by international benchmarks for genomic medical research.

### Supercomputers dedicated to medical and life sciences

#### Computer architectures and software

Since the start of the Human Genome Project, supercomputers specialized for large-scale genome analysis have been maintained and operated in Japan. Among them, the following are widely open to general life and medical researchers, including those outside their institutes: the ToMMo supercomputer at the Tohoku Medical Megabank Organization^21^ at Tohoku University, SHIROKANE^21^ at the Human Genome Center (HGC) of the Institute of Medical Science at the University of Tokyo and the NIG supercomputer^22,23^ at the National Institute of Genetics (NIG). Although the NIG supercomputer is primarily used for the construction and operation of the INSDC databases, as described later, it also provides computational resources to general researchers, as is the case with SHIROKANE and the ToMMo supercomputer.

In general, the performance requirements of a computer can be summarized as the following characteristics for each application area of computing^24,25^.

- Computer simulation: Double-precision floating-point arithmetic performance is important, as is the ability to perform distributed memory jobs. The output should be greater than the input, and the output is rarely read.
- Deep learning: Lower precision floating-point arithmetic performance is important, and an inference can be 8-bit (GPGPU/TPU). Input is more important than output; training data are reused.
- Big data analysis: Integer arithmetic performance is important along with double-precision floating-point arithmetic performance. Database (DB) utilization includes MapReduce/SPARK. The ability to process a large number of independent jobs is necessary. Large-data input and output are both important, and output is often reused.

The NIG supercomputer is a cluster computer designed for large-scale genome analysis^23,24^. In a cluster computer, all computers (called nodes) are used in parallel to accelerate computation speed. Each node and large-capacity storage are interconnected by a high-speed network (InfiniBand) to accelerate data transfer and storage read/write operations in parallel. In general, genome analysis tends to require relatively large memory capacities. In addition to distributed memory computational nodes with 8 GB per CPU core (thin nodes), the NIG supercomputer has computation nodes with 3 TB of shared memory per node (medium node) and 12 TB of shared memory node (fat node).

The peak performance of the NIG supercomputer is 1.1 PFLOPS (CPU: 599.8 TFLOPS, GPGPU: 499.2 TFLOPS), the total memory capacity is 138.8 TB, and the total storage capacity is 43.8 PB. The HPC cluster includes 16 GPGPU nodes that have four GPGPUs (NVIDIA Tesla V100 SXM2) for each chassis. The NIG supercomputer is currently connected to SINET6 with a theoretical bandwidth of 30 Gbps (theoretically capable of transferring 1 PB of data in 24 h), which will be expanded to 100 Gbps in the future. As a result, high-speed communication with universities and research institutions nationwide is now possible^23,24^.

Basically, the NIG supercomputer allocates the computing resources of the bare-metal servers to users using the grid engine^26^ and Slurm^27,28^ task managers for the sake of simplicity. This is a traditional method used in high-performance computing and is different from the method used in commercial cloud computing, which uses elaborate dashboards and APIs to allocate resources, such as virtual machines. It was selected because the simple task manager-based approach also simplifies construction and reduces operation costs.

To enable large-scale personal genome analysis, the inside network of the NIG supercomputer is divided into two independent network sections: a general genome analysis environment (general analysis section) and a newly established personal genome analysis section with a high level of security. The personal genome analysis section was established to prevent researchers from peeking at parts of each other’s computational work on shared computers. For this reason, the personal genome analysis section is rented on a node-by-node or virtual machine-by-virtual machine basis.

It has been indicated that differences in supercomputer architecture and usage make it difficult to ensure the reproducibility of analysis and secondary use data. To address this problem, efforts are underway to standardize workflow engines and standardize virtual or real computational environments by using container virtualization (especially Singularity CE and Apptainer^29,30^) or package managers that can be used on shared computers with user privileges (e.g., Speck, Supercomputer Package Manager^31^, GNU Guix^32^, and Conda Package Manager).

#### User registration

User registration for the NIG supercomputer is accepted at any time. Since the supercomputer system is subject to export control regulations under the Foreign Exchange and Foreign Trade Law (Foreign Exchange and Foreign Trade Act^33^), the person responsible for user registration must be a resident of Japan and a faculty member of a university or national/public research institute, as stipulated by the Foreign Exchange and Foreign Trade Law. However, while researchers from private companies and overseas researchers cannot register directly, they can still use the system in collaboration with registered users.

#### Genomic data sharing on supercomputers

There are two storage systems in the NIG supercomputer: the storage area for the calculation suitable for I/O intensive tasks (Lustre file system, 17.1 PB in total) and the deoxyribonucleic acid (DNA) data archive, which is constructed as a hierarchical storage system with 15 PB hard disk drive (HDD)-based storage and a 15 PB tape library system. The former is mainly used as storage for analytical calculations, while the latter is used to store INSDC data.

In recent years, to meet increasing demands for quick and easy sharing of personal genomic data within research groups, the NIG supercomputer has provided a service that installs data sharing middleware on rented compute nodes in its personal genome analysis section of the NIG supercomputer. For example, by combining a Linux remote desktop environment (users can also use the Ubuntu Linux desktop environment via a web browser) and data sharing middleware compatible with the Amazon S3 protocol, users can readily share and analyze data using the supercomputer without taking any data out of the NIG supercomputer.

The main purpose of the NIG supercomputer is to provide the computational infrastructure for INSDC, which was jointly constructed by the DNA Data Bank of Japan (DDBJ), the National Center for Biotechnology Information (NCBI) in the US, and the European Biometric Institute (EBI). For that reason, INSDC contains all DNA sequence data that can be referenced from papers, and since data are exchanged daily within the three regions, three official mirrors exist in Japan, the US and Europe^34^.

Here, it should be noted that since the start of the Sequence Read Archive (SRA) in 2008, the amount of data has increased rapidly and is now several tens of thousands of times larger than before, which has led to concerns that the inability to store rapidly growing data could cause the INSDC data archiving scheme to fail. However, data volume growth has been converging and has been constant at ~1.3 times per year since 2014^35^, which is not considerably different from the growth in capacity per unit storage cost, so the current on-premise-based method is expected to remain stable in the immediate future. Furthermore, if the growth of data continues to slow, temporary measures can be expected to remain effective.

Nevertheless, even if data volume increases are reduced, problems will eventually occur if data volume and capacity growth are not balanced, which means that database archiving could fail in the not-too-distant future. Although this issue calls to mind a period at the beginning of the SRA construction when the growth of the data volume was two to six times per year, it should also be remembered that the DDBJ overcame that data growth problem by increasing the performance and storage capacity of the supercomputer by more than 15 times. This was accomplished by drastically changing the NIG supercomputer’s internal software systems and hardware configuration^36^.

## Use of commercial clouds

The continuing deluge and ever-expanding use of genomic data have led to a rapid increase in demands for networks, storage systems, and computing resources. In our current era, large-scale genomic data analysis is no longer limited to specialized institutions but is now being conducted in single laboratories, as well. To meet the rapidly growing demands of computation and to promote the concept of “bringing people to the data”, commercial clouds, such as Amazon Web Service (AWS), Google Cloud Platform (GCP), and Microsoft Azure are becoming indispensable due to their ability to provide and manage vast amounts of computer resources.

One of the major advantages of a commercial cloud is elasticity, which enables users to rent and pay for the exact resources needed. That, in turn, allows for small to quite large projects to be accommodated without difficulty. Another major advantage of commercial cloud computing is that it enables international data sharing, a shared analysis workflow, and analysis reproducibility. In the case of an international genome analysis project that is conducted within a traditional computation environment, data are normally downloaded and analyzed at each analysis site. In contrast, a commercial cloud allows data to be brought to a single location for analysis. In addition, major cloud providers offer their computing environments in units called instances, and the same type of instance can be used all over the world, which makes it easy to guarantee the reproducibility of the analysis^37^.

Although commercial cloud computing addresses many problems posed by genomic data sharing, a particular concern in medical data analysis is security, which means researchers need to follow various security guidelines. However, in many cases, it is not easy for each laboratory to prepare computing resources that meet these guidelines, so commercial clouds are often certified to various security standards. These certifications provide a good starting point for preparing computing infrastructures.

**Fig. 2:**
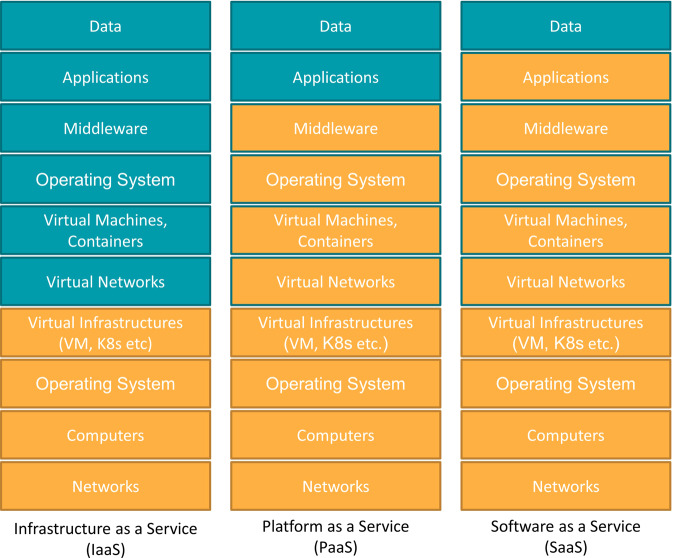
Responsibility demarcation points of the cloud computing services. Blue is the user’s area of responsibility and orange is the cloud provider’s area of responsibility. Cloud computing is characterized by multi-layered, with virtual networks and computers running on top of physical networks and computer systems.

It should be noted that not all activities performed on cloud computing infrastructures are the responsibility of the cloud vendor, and it is important to understand where the boundary of responsibility lies in relation to the services being used (Fig. 2). Cloud services are often broadly classified into the following three categories: Infrastructure as a Service (IaaS), defined as services that lend out computing infrastructure, such as AWS, GCP, and Microsoft Azure; Platform as a Service, which are services that provide an environment for programmers, such as Salesforce, Heroku, and VMware Tanzu; and Software as a Service (SaaS), which are services that directly provide application software, such as Gmail.

The most common form of cloud computing is currently IaaS. In traditional computing infrastructures, a responsibility demarcation point between the user and the infrastructure engineer is normally found around the middleware and is said to cause high communication costs and low development efficiency in program development. In contrast, the virtual infrastructure technology of commercial clouds allows users to control the lower layers, including virtual machines and virtual networks. This means that users need broader skill sets in the age of cloud computing. There is also a need for cloud-native system design techniques that consider system configurations from the beginning to conserve commercial cloud usage fees and provide efficient security measures for cloud infrastructures.

### Laws, certifications, and security guidelines

This section describes laws and guidelines related to cloud security, certification systems, personal information protection, and computer crimes that should be considered when conducting personal genome analysis through the use of cloud environments in Japan. The Act on the Protection of Personal Information and related guidelines, including those of foreign countries, were introduced in a previous article^38^. Below, related security laws and guidelines are discussed.

As part of the government of Japan’s cybersecurity efforts, The Basic Act on Cybersecurity, which was enacted in December 2014, stipulates that a basic plan for cybersecurity (cybersecurity strategy) must be established.

In accordance with that law, Cybersecurity Strategy 2015 was published in September 2015. It has since been translated into English^39^ and is expected to be read by foreign government organizations. The strategy consists of three axes: security and crisis management, economy, and protection of citizens and users. The strategy is updated every three years^40^.

Since the Cybersecurity Strategy states that each government agency will implement cybersecurity measures based on unified standards, Japan’s National Center of Incident Readiness and Strategy for Cybersecurity (NISC) has published “Common Standards Group for Cybersecurity Measures by Government Agencies and Related Agencies“^41^.

In the 2021 version of that publication, a description was added that anticipates the increased use of cloud services in addition to the need for a zero-trust architecture to address situations where sufficient security cannot be guaranteed by perimeter-based defenses alone. The publication also states that Japan’s newly established digital agency (DA) will develop and operate the systems commonly used by the government in cooperation with each government ministry and agency and that cloud services (government cloud) will be developed in stages.

Separately, in 2017, the Federation of Economic Organizations (Keidanren) released a “Proposal Calling for Enhanced Cybersecurity“^42^, which stated that even though Japan’s cybersecurity strategy is formulated centrally by NISC, unification is needed because specific policies are implemented separately by each ministry. Therefore, it is necessary to understand the two lines of Japanese security policy: the independent efforts of each ministry and the centralized efforts of the government.

To integrate all of the above into an understandable package, the Japan Institute for Promotion of Digital Economy and Community (JIPDEC)^43^ has prepared a list of laws, regulations, and guidelines related to cloud services in Japan^44,45^. Tables 1 through 3 provide summaries of the items closely related to large-scale genome analyses on cloud computing infrastructures in Japan.

**Table 1:** Security Guidelines and Certification Systems.

ISMS Cloud Security Certification https://isms.jp/english/index.html	ISMS Accreditation Center (ISMS-AC)	Providers Customers	Assuming ISMS certification, ISO/IEC 27017 control measures will be added to ISO/IEC 27001 control measures.
Cloud Security Mark https://www.jasa.jp/en/	Japan Information Security Audit Association	Providers	The basic statement requirements are defined in the Cloud Information Security Management Standard based on the “Information Security Management Guidelines for the Use of Cloud Services” published by METI.
Information system Security Management and Assessment Program (ISMAP) https://www.ismap.go.jp/csm	Digital Agency, MIC, METI, and IPA	Providers	In principle, government agencies will procure services from those listed on the ISMAP Cloud Services List.
Common Standards for Cybersecurity Measures for Government Agencies and Related Agencies https://www.nisc.go.jp/eng/pdf/kijyunr3-en.pdf	NISC	Customers (Government agencies)	Based on the Cyber Security Basic Law (Law No. 104 of 2014), the standards for measures related to cybersecurity for national administrative agencies, etc., are determined.
Information Security Guidelines for Cloud Service https://www.soumu.go.jp/main_sosiki/joho_tsusin/eng/pressrelease/2021/9/30_06.html	MIC	Providers	Guidelines outlining information security measures that cloud providers should implement.
Guideline for the Security Management of Medical Information Systems, Version 5.2 https://www.mhlw.go.jp/stf/shingi/0000516275_00002.htm	MHLW, METI and MIC	Customers (Healthcare professionals)	
Guidelines for Safety Management of Medical Information by Providers of Information Systems and Services Handling Medical Information https://www.meti.go.jp/policy/mono_info_service/healthcare/teikyoujigyousyagl.html	MHLW, METI and MIC	Providers

**Table 2:** Act on Information Protection.

Name of law	Main reference clauses
The Constitution of Japan	Article 21
Act on the Protection of Personal Information	General
Act for Establishment of the Information Disclosure and Personal Information Protection Review Board	General
Act on Arrangement of Relevant Acts Incidental to Enforcement of Act on the Protection of Personal Information Held by Administrative Organs	General
Act on Regulation, etc. of Loan Business	Article 30(2): Prohibition of Use for Unintended Purpose of Personal Credit Data
Installment Sales Act	Article 39 Proper Use of Credit Information
Unfair Competition Prevention Act	General

**Table 3:** Acts related to computer crimes.

Name of law	Main reference clauses
Penal Code	Article 7-2: Definitions of the Electronic or Magnetic Record
	Article 157(1): False Electronic or Magnetic Record in the Original of Notarized Deeds
	Article 158(1): False Record of a Electromagnetic Notarial Act Original Service Crime
	Article 161-2(1): Crime of Unauthorized Creation and Use of Electromagnetic Records
	Article 234-2: Crime of Obstruction of Business by Damaging a Computer
	Article 246-2: Crime of Computer Fraud
	Article 258: Crime of Damaging a Document or an Electronic or Magnetic Record
	Article 259: Crime of Damaging a Private Document or Electronic or Magnetic Record
Act on Prohibition of Unauthorized Computer Access	General
Labor Standards Act	Article 91: Restrictions on Provisions for Sanctions
Act for Securing the Proper Operation of Worker Dispatching Undertakings and Improved Working Conditions for Dispatched Workers	General
Act for Establishment of the Information Disclosure and Personal Information Protection Review Board	Article 24-4: Obligation to Observe Secrecy
Act on Regulation and Punishment of Acts Relating to Child Prostitution and Child Pornography, and the Protection of Children	General

The security measures to be taken by research institutes that conduct large-scale genome analyses are described in the “Guidelines for the Safety Management of Medical Information Systems”. These guidelines basically take up ISO/IEC 27001 as a standard information security management system (ISMS) that requires appropriate security management throughout organizations to ensure compliance.

If the affiliated organization is a government organization, it is also necessary to refer to the “Common Standards”. When government agencies, etc., use cloud services, it is necessary to satisfy each of the compliance requirements described in the “outsourcing” section. In particular, the Common Standards state that the criteria for selecting an external service provider when using cloud services must satisfy the information system security management and assessment program (ISMAP)^46,47^, as well as provisions equivalent to the “Criteria for Selecting Outsourcees.”

The “Guidelines for Safety Management of Medical Information by Providers of Information Systems and Services Handling Medical Information” were compiled by the Ministry of Health, Labor and Welfare (MHLW), the Ministry of Internal Affairs and Communications (MIC), and the Ministry of Economy, Trade and Industry (METI) to define the security guidelines required for cloud providers who handle medical data and describe how to set responsibility demarcation points with users. It also provides detailed descriptions of security risk management procedures.

## Implementation of multiclouds and hybrid clouds

Commercial cloud services are prone to oligopoly due to economies of scale, and even in Japan, a small number of major cloud providers (AWS, GCP, Microsoft Azure) hold a high market share. In retrospect, it can be argued that some of the main advantages of the commercial clouds described in the previous section are the result of this oligopoly.

In June 2022, The Japan Fair Trade Commission (JFTC) released a “Report on the State of Trade in the Cloud Services Sector”^48^, in which they clarified the state of trade and competition in the cloud services sector, as well as their thoughts on antitrust law and competition policy. The report pointed out the following about commercial clouds:

- The use of cloud services is currently increasing, and under this type of market environment, it is relatively more important for cloud service providers to acquire new users than to retain them. Therefore, cloud service providers currently have the incentive to compete for new users in terms of price and quality.
- Although there are indications of a certain degree of competition at present, the market is likely to change to a noncompetitive structure in the future due to the high market share already held by a small number of operators and the cost of switching services, which hinders switching to other services.
- If the cloud services market changes to a noncompetitive structure and there is no competitive pressure from neighboring service sectors, the competitive pressure on existing cloud service providers will weaken. This could have a negative impact on the quality of cloud services, including security, data processing, loading speeds, and connectivity between functions, as well as on the transparency of transaction terms and conditions for users. While the prices of cloud services are currently on a general downward trend, there are indications that some services are raising their prices at a time when it is becoming difficult to diplomatize with users^49^.
- In the cloud services market, it is important to ensure an environment in which users can freely select services that will prevent the adverse effects that can result from the concentration of market share, as well as to improve service quality and cost proposals through competition. However, the share of the three major cloud providers (AWS, GCP, and Azure) in Japan is currently 70%, and only ~15% of the current cloud operators have realized a multicloud environment. The report also indicates that many Japanese users do not feel the need to move to multiple clouds at this time, as 70% of all users have not experienced any problems with their current use of cloud computing.

Other general concerns regarding the use of commercial cloud computing include the following: (1) Unexpected costs: It is easy to forget the cost of computing instances for development and testing and the network costs for mass downloads^50^. (2) Data management policy issues: Some companies may want to keep their data in-house, and there are issues related to restrictions on the transfer of personal information outside the region. (3) Network latency issues: Communication delays due to the need for network communication with geographically distant locations^50^. (4) Concerns about political policies for commercial clouds: There are political concerns about the dangers of relying on foreign companies for core infrastructure^51^. (5) Public understanding issues: This includes the problem of public understanding regarding the payment of large amounts of the country’s budget to foreign companies.

### Implementation with commercial clouds and software products

To facilitate large-scale genome analysis, it would be ideal if computational resources managed by multiple laboratories, including on-premise and multiple commercial cloud vendors, could be integrated to create a single computing environment. However, there are substantial technical difficulties in this regard, particularly around identity management and secure authentication. In theory, it would be possible to approach this ideal goal by, for example, combining Linux containers and virtual network technologies, and it would be very helpful to have some kind of commercial service or product to actually operate the system and be used in the analysis.

One hybrid cloud service that is currently available as a commercial cloud service is Azure Arc. This dedicated system, which is designed for the purpose of connecting Microsoft Azure to on-premise or other commercial clouds in order to mediate control, can even offload Microsoft Azure workloads to the outer computational resources^52^. A similar configuration can be realized by using Kubernetes with commercial support. Note that Kubernetes is a portable open-source platform for managing containerized workloads and services^53^. One example of such a system is the HPE Ezmeral Runtime Enterprise^54^, which can manage multiple Kubernetes clusters. These Kubernetes clusters can be located in on-premise compute environments or on commercial clouds. If these Kubernetes clusters can communicate with each other using a virtual private network, they can be used as a single, integrated computing environment.

## Conclusion

This mini-review has described the current status of shared computers available for genome analysis in Japan while also providing an overview of the current Japanese laws and systems for cloud computing infrastructures. It was written to address the urgent need to promote “bringing people to the data” through the use of cloud environments in response to the rapid spread of next-generation sequencing (NGS) analysis, the expansion of international data sharing, and trends in international data analysis research.

However, the Japanese commercial cloud marketplace is currently dominated by US commercial cloud vendors, which account for more than 70% of the market, and the market share of Japanese domestic vendors is showing a declining trend, which makes it difficult for Japanese government agencies, universities, and research organizations to actively utilize commercial cloud services. Furthermore, while the government is promoting the use of cloud computing to improve security, availability, and cost performance, it is also carefully considering the potential for adverse effects. In fact, the government is planning to implement a dedicated government cloud in a multicloud environment in which domestic vendors are expected to participate gradually.

Although multicloud and hybrid cloud computing are currently difficult to implement due to unresolved technical difficulties, such as authentication, some commercial services and products have already emerged, making it feasible to build some functions, and further research and development are expected.

## References

[1] Van der Auwera, G. & O’Connor, B. *Genomics in the Cloud*. (O’Reilly Mediam, 2020).

[2] NVIDIA Clara Parabricks, https://www.nvidia.com/en-us/clara/genomics/, Accessed 11 December 2022.

[3] McKenna A (2010). The Genome Analysis Toolkit: a MapReduce framework for analyzing next-generation DNA sequencing data. Genome Res.

[4] DePristo MA (2011). A framework for variation discovery and genotyping using next-generation DNA sequencing data. Nat. Genet.

[5] Zhao, S., Agafonov, O., Azab, A., Stokowy, T. & Hovig, E. Accuracy and efficiency of germline variant calling pipelines for human genome data. *bioRxiv* https://www.biorxiv.org/content/10.1101/2020.03.27.011767v1 (2020). 10.1038/s41598-020-77218-4 PMC7678823 33214604

[6] Freed, D., Aldana, R., Weber, J. A. & Edwards, J. S. The Sentieon Genomics Tools—a fast and accurate solution to variant calling from next-generation sequence data. *bioRxiv* https://www.biorxiv.org/content/10.1101/115717v2 (2017).

[7] PEZY Computing, News: Whole human genome analysis completed in less than 15 minutes (2020), https://www.pezy.co.jp/news/20201023/, Accessed 11 December 2022.

[8] Ebisuzaki. *The development of the application software for PEZY SC2 many core processors*. *A: Barcelona Supercomputing Center*. https://bsc.es/sites/default/files/public/u2416/bsc1.pdf (2020).

[9] The Research Organization for Information Science and Technology (RIST), HPCI: High Performance Computer Infrastructure*,* https://www.hpci-office.jp/folders/english, Accessed 11 December 2022.

[10] Top500 Organization, Top500 List, https://www.top500.org/, Accessed 11 December 2022.

[11] HPCG Organization, HPCG benchmark Results, https://www.hpcg-benchmark.org/, Accessed 11 December 2022.

[12] Graph500 Organization, The Graph500 List, https://graph500.org/, Accessed 11 December 2022.

[13] Ueno K, Suzumura T, Maruyama N, Fujisawa K, Matsuoka S (2017). Efficient breadth-first search on massively parallel and distributed memory machines. Data Sci. Eng..

[14] Ueno, K., Suzumura, T., Maruyama, N., Fujisawa, K. & Matsuoka, S. Extreme scale breadth-first search on supercomputers. In *2016 IEEE International Conference on Big Data (Big Data), IEEE*; 1040–1047 (2016).

[15] HPL-MxP Organization, HPL-AI (HPL-MxP) Results, https://hpl-mxp.org/results.md, Accessed 11 December 2022.

[16] Matsuoka, S. Fugaku A64FX: the first exascale supercomputer and its innovative Arm CPU. In *2021 Symposium on VLSI Circuits*, IEEE (2021).

[17] National Institute of Informatics, SINET6, https://www.sinet.ad.jp/en, Accessed 11 December 2022.

[18] Kurimoto, T. et al. SINET5: a low-latency and high-bandwidth backbone network for SDN/NFV Era. In *IEEE International Conference on Communications (ICC)*, IEEE (2017).

[19] Internet2, https://internet2.edu/, Accessed 11 December 2022.

[20] GÉANT Network, https://network.geant.org/, Accessed 11 December 2022.

[21] Tohoku University, Tohoku Medical Megabank Organization, https://www.megabank.tohoku.ac.jp/english/, Accessed 11 December 2022.

[22] The Institute of Medical Science, The University of Tokyo, SHIROKANE supercomputer, https://gc.hgc.jp/en/, Accessed 11 December 2022.

[23] National Institute of Genetics, the NIG supercomputer, https://sc.ddbj.nig.ac.jp/en/, Accessed 11 December 2022.

[24] Ogasawara O, Kodama Y, Mashima J, Kosuge T, Fujisawa T (2020). DDBJ database updates and computational infrastructure enhancement. Nucleic Acids Res.

[25] Wozniak, J. *An introduction to scalable deep learning workflows with CANDLE. CANDLE Workshop*. https://wiki.nci.nih.gov/display/HPC/CANDLE+Workshops?preview=/357701616/362972918/Day%202%20PM%20-%20Wozniak.pdf (2018)

[26] Altair Engineering Inc., Altair Grid Engine*,* https://www.altair.com/grid-engine/, Accessed 11 December 2022.

[27] SchedMD LLC, Slurm Workload Manager, https://slurm.schedmd.com/overview.html, Accessed 11 December 2022.

[28] Andy, B. Y., Jette, M. A. & Grondona, M. Slurm: Simple linux utility for resource management. In *Workshop on job scheduling strategies for parallel processing*, Springer, Berlin, Heidelberg, 44–60 (2003).

[29] Kurtzer GM, Sochat V, Bauer MW (2017). Singularity: scientific containers for mobility of compute. PLoS ONE.

[30] The Apptainer Project, https://apptainer.org/, Accessed 11 December 2022.

[31] Gamblin T (2015). The spack package manager: bringing order to HPC software chaos. Int. Conf. High. Perform. Comput. Netw. Storage Anal. SC 2015.

[32] Ludovic C (2013). Functional package management with Guix. arXiv Prepr. arXiv.

[33] Ministry of Justice, Foreign Exchange and Foreign Trade Act. https://www.japaneselawtranslation.go.jp/en/laws/view/3700 (2022).

[34] Karsch-Mizrachi I, Takagi T, Cochrane G (2018). The International Nucleotide Sequence Database Collaboration. Nucleic Acids Res..

[35] Cook CE, Stroe O, Cochrane G, Birney E, Apweiler R (2020). The European Bioinformatics Institute in 2020: building a global infrastructure of interconnected data resources for the life sciences. Nucleic Acids Res..

[36] Ogasawara O (2013). DDBJ new system and service refactoring. Nucleic Acids Res..

[37] Langmead B, Nellore A (2018). Cloud computing for genomic data analysis and collaboration. Nat. Rev. Genet..

[38] Tanjo T, Kawai Y, Tokunaga K, Ogasawara O, Nagasaki M (2021). Practical guide for managing large-scale human genome data in research. J. Hum. Genet..

[39] National center of Incident readiness and Strategy for Cybersecurity (NISC), Cybersecurity Strategy, https://www.nisc.go.jp/eng/pdf/cs-strategy-en.pdf, Accessed 11 December 2022.

[40] National center of Incident readiness and Strategy for Cybersecurity (NISC), Outline of the Cybersecurity Strategy, https://www.nisc.go.jp/eng/index.html#sec2, Accessed 11 December 2022.

[41] National center of Incident readiness and Strategy for Cybersecurity (NISC), Common Standards for Information Security Measures for Government Agencies (FY2016),https://www.nisc.go.jp/eng/archive.html, Accessed 11 December 2022.

[42] KEIDANREN, Calls for Enhanced Cybersecurity to Achieve Society 5.0, https://www.keidanren.or.jp/policy/2017/103.html, Accessed 11 December 2022.

[43] JIPDEC,https://english.jipdec.or.jp/index.html, Accessed 11 December 2022.

[44] JIPDEC, Introduction of domestic and overseas systems and guidelines related to cloud services, https://www.jipdec.or.jp/library/JIP-ISMS201-1.1.html, Accessed 11 December 2022.

[45] JPIDEC, ISMS User’s Guide for Healthcare Organizations*,* https://www.jipdec.or.jp/archives/publications/JIP-ISMS114-21.pdf, Accessed 11 December 2022.

[46] Cabinet Secretariat, Ministry of Internal Affairs and Communications (MIC), and Ministry of Economy, Trade and Industry (METI), ISMAP Overview*,* https://www.ismap.go.jp/sys_attachment.do?sys_id=927d7c80dbdfd9506e6cb915f39619c8, Accessed 11 December 2022.

[47] Cabinet Secretariat, Ministry of Internal Affairs and Communications (MIC), and Ministry of Economy, Trade and Industry (METI), ISMAP Cloud Service List, https://www.ismap.go.jp/csm?id=cloud_service_list, Accessed 11 December 2022.

[48] The Japan Fair Trade Commission, Report on the State of Trade in the Cloud Services Sector, https://www.jftc.go.jp/houdou/pressrelease/2022/jun/220628.html, Accessed 11 December 2022.

[49] Cloud Infrastructure Services Providers in Europe (CISPE), Cloud Infrastructure Services: an analysis of potentially anti-competitive practices, https://cispe.cloud/studies/, Accessed 11 December 2022.

[50] Pritchard, S., Cloud repatriation: five reasons to repatriate data from cloud*,**ComputerWeekly.com* https://www.computerweekly.com/feature/Cloud-repatriation-Five-reasons-to-repatriate-data-from-cloud, 17 November 2021.

[51] Merkel Calls Trump Ban from Twitter, Other Media Platforms ‘Problematic’, *Voice of America News,* https://www.voanews.com/a/europe_merkel-calls-trump-ban-twitter-other-media-platformsproblematic/6200592.html, 11 January 2011.

[52] Malik, A. & Kaur, D. *Implementing Hybrid Cloud with Azure Arc*. (Packet Publishing, 2021).

[53] The Linux Foundation, Kubernetes, https://kubernetes.io/docs/concepts/overview/, Accessed 11 December 2022.

[54] Hewlett Packard Enterprise Company, HPE Ezmeral Runtime Enterprise, https://www.hpe.com/us/en/software/ezmeral-runtime.html, Accessed 11 December 2022.

